# Mice Deficient in Epithelial or Myeloid Cell Iκκβ Have Distinct Colonic Microbiomes and Increased Resistance to *Citrobacter rodentium* Infection

**DOI:** 10.3389/fimmu.2019.02062

**Published:** 2019-09-10

**Authors:** Amy R. Mackos, Jacob M. Allen, Eunsoo Kim, Chris A. Ladaika, Raad Z. Gharaibeh, Cathy Moore, Nicola M. A. Parry, Prosper N. Boyaka, Michael T. Bailey

**Affiliations:** ^1^Center for Microbial Pathogenesis, The Research Institute at Nationwide Children's Hospital, Columbus, OH, United States; ^2^Department of Veterinary Biosciences, The Ohio State University, Columbus, OH, United States; ^3^Department of Bioinformatics and Genomics, University of North Carolina at Charlotte, Charlotte, NC, United States; ^4^Bioinformatics Services Division, Department of Bioinformatics and Genomics, University of North Carolina at Charlotte, Kannapolis, NC, United States; ^5^Division of Comparative Medicine, Massachusetts Institute of Technology, Cambridge, MA, United States; ^6^Department of Pediatrics, College of Medicine, The Ohio State University, Columbus, OH, United States

**Keywords:** *Citrobacter rodentium*, NF-κB, colonic epithelial cells, microbiota, colitis

## Abstract

The colonic microenvironment, stemming from microbial, immunologic, stromal, and epithelial factors, serves as an important determinant of the host response to enteric pathogenic colonization. Infection with the enteric bacterial pathogen *Citrobacter rodentium* elicits a strong mucosal Th1-mediated colitis and monocyte-driven inflammation activated via the classical NF-κB pathway. Research has focused on leukocyte-mediated signaling as the main driver for *C. rodentium*-induced colitis, however we hypothesize that epithelial cell NF-κB also contributes to the exacerbation of infectious colitis. To test this hypothesis, compartmentalized classical NF-κB defective mice, via the deletion of IKKβ in either intestinal epithelial cells (IKKβ^ΔIEC^) or myeloid-derived cells (IKKβ^ΔMY^), and wild type (WT) mice were challenged with *C. rodentium*. Both pathogen colonization and colonic histopathology were significantly reduced in IKKβ-deficient mice compared to WT mice. Interestingly, colonic IL-10, RegIIIγ, TNF-α, and iNOS gene expression were increased in IKKβ-deficient mice in the absence of bacterial challenge. This was associated with increased p52, which is involved with activation of NF-κβ through the alternative pathway. IKKβ-deficient mice also had distinct differences in colonic tissue-associated and luminal microbiome that may confer protection against *C. rodentium*. Taken together, these data demonstrate that classical NF-κB signaling can lead to enhanced enteric pathogen colonization and resulting colonic histopathology.

## Introduction

Gastrointestinal infections are a significant source of morbidity and mortality worldwide with enterohemorrhagic *Escherichia coli* (EHEC) and enteropathogenic *E. coli* (EPEC) contributing significantly to bacterial-induced gastrointestinal disease. As members of the attaching and effacing (A/E) family, these Gram negative bacteria must intimately attach to intestinal epithelial cells to inject effector molecules via a type III secretion system resulting in actin-rich pedestal formation (1, 2). Virulence factors are encoded within the locus of enterocyte effacement, a pathogenicity island with shared sequence homology between family members. Pathogenic EPEC and EHEC do not readily colonize mice. Therefore, *Citrobacter rodentium*, a naturally occurring murine pathogen and member of the A/E family, is used to study EPEC/EHEC host responses (3–5). Inoculation with *C. rodentium* leads to distal colon colonization, with the peak of colonization occurring between Days 10 and 14 post challenge and full clearance by Day 24 post-challenge in wild type mice. *C. rodentium* colonizes all mouse strains, however the degree of colonization and resulting colitis severity is dependent on host genetics and intestinal microbial composition (5–8). Mice challenged with *C. rodentium* develop Th1-mediated infectious colitis characterized by inflammatory monocyte/macrophage and neutrophil accumulation (3–6). The response to *C. rodentium* infection is initiated by colonic epithelial cells (CECs) and is perpetuated by newly recruited immune cells including inflammatory monocytes, neutrophils, and Th1 and Th17 CD4^+^ T cells through the activation of transcription factors, such as NF-κB (3, 4, 6, 9).

NF-κB transcription factors belong to an evolutionarily conserved family that is ubiquitous to virtually all mammalian cells, which upon activation results in the rapid transcription of genes involved in immunity and inflammation (10). Activation can occur via two distinct pathways: classical (canonical) and alternative (non-canonical). Classical activation is dependent on the degradation of inhibitor of κB (IκB), that sequesters NF-κB in the cytoplasm during homeostasis. Traditionally the classical pathway is activated by bacterial and viral antigens through Toll-like receptor ligation, proinflammatory cytokines i.e., IL-1β and TNF-α, or oxidative stress. Regardless of the stimulus, a signaling cascade results in the activation of the inhibitor of κB kinase (IKK) which consists of three subunits: IKKα, IKKβ, and IKKγ, with IKKα and IKKβ serving as catalytic subunits and IKKγ serving as the catalytic subunit regulator (11). During classical activation, IKKγ activates IKKβ which phosphorylates IκB, causing a conformational change to release NF-κB subunits. The newly free NF-κB, i.e., p65/p50, translocate to the nucleus where they bind κB-specific response elements in promoter regions of various target genes, including proinflammatory cytokines, e.g., TNF-α, IFN-γ, and IL-1β, chemokines, e.g., CCL2, CXCL1, CX3CL1, and effector molecules, e.g., iNOS and β-defensin 2 (10, 11). Just as the induction of NF-κB is of great importance to clear infection, its deactivation is also necessary to prevent an overzealous inflammatory immune response which can lead to tissue destruction. This is further highlighted by the fact that NF-κB actively transcribes its own inhibitor, IκB, as a feedback mechanism.

*Citrobacter rodentium* infection leads to the activation of classical p65/p50 and p50/p50 NF-κB dimers that parallels pathogenic colonization (12). Activation can be observed as early as 1 Day post-challenge in CECs and 5 Days post-challenge in the lamina propria (12, 13). During *C. rodentium* challenge, NF-κB activation begins with the ligation of pattern recognition receptors which recognize conserved bacterial motifs such as LPS, flagellin, and peptidoglycan. NF-κB activation is necessary for bacterial clearance; however, uncontrolled activation can lead to an overzealous host response, including excessive production of chemokines necessary for enhanced immune cell recruitment, leading to overproduction of proinflammatory cytokines that contributes to tissue damage (9). While much of the research pertaining to *C. rodentium* and its A/E family members has focused on the response of lymphoid and myeloid cells, there is evidence that CECs are significant contributors to inflammation and immune cell recruitment in response to pathogen challenge (14, 15). Additionally, proinflammatory cytokines (e.g., TNF-α and IL-1β), chemokines (e.g., CCL2 and CXCL1), and enzymes (e.g., iNOS), are up-regulated in CECs and newly recruited immune cells as a result of signaling cascades which coalesce with, and result from, NF-κB activation (9, 13, 16, 17). Thus, this study was designed to test the hypothesis that epithelial-induced NF-κB-derived activation is necessary for infectious colitis exacerbation via inflammatory monocyte recruitment. This study also addressed the corollary hypothesis that inflammatory monocytes contribute to infectious colitis via NF-κB activation. To test this hypothesis, two different strains of mice were challenged with *C. rodentium* in which classical NF-κB activation is defective via the deletion of IKKβ in either intestinal epithelial cells (IKKβ^ΔIEC^) or myeloid-derived cells (IKKβ^ΔMY^).

## Materials and Methods

### Mice

Male C57BL/6 mice aged 6–8 weeks served as wild type (WT) controls were purchased from Charles River Laboratories (Wilmington, MA). Two different strains of IKKβ-deficient mice were obtained from Dr. Prosper Boyaka, in which IKKβ was selectively eliminated in all myeloid-derived cells (IKKβ^ΔMY^) or in intestinal epithelial cells (IKKβ^ΔIEC^). Briefly, mice were originally generated via crossing mice with a *lox*P-flanked IKKβ gene (*Ikbkb*^f/f^) with a mouse with *Cre* downstream of either the lysosome promoter (LysMCre) for IKKβ^ΔMY^ mice or downstream of the villin promoter (Villi-Cre) for IKKβ^ΔIEC^ (18–21). All mice were housed in the same vivarium, and allowed to acclimate for at least 1 week prior to experiments as previously described (22, 23).

### Infection and Bacterial Enumeration

*Citrobacter rodentium* strain DBS120 (pCRP1::Tn5) (24) was grown as previously described (22). Mice were challenged via oral gavage with 100 μl PBS containing 3 × 10^8^ CFU. The day of infection is referred to as day 0 and all data collected will be referenced as Days post-challenge. *C. rodentium* colonization was monitored on Days 0, 3, and 12 post-challenge. Fresh stool was collected directly from the colon at the time of euthanasia and grown on MacConkey agar supplemented with kanamycin (40 μg/ml) to quantify *C. rodentium* load as described previously (22).

### Histopathology

Colons were fixed in 10% formalin-buffered phosphate, embedded in paraffin, and stained with hematoxylin and eosin (H&E) for histopathological evaluation by a blinded, board-certified veterinary pathologist (N.M.A.P.). The severity of colonic lesions was scored, according to previously defined criteria (25). Each colon was scored on six different categories; inflammation, dysplasia, hyperplasia, edema, crypt defects, and epithelial defects. Each category received a score of 0 to 4 in 0.5 increments based on the degree of lesion severity: 0 (absent), 1 (mild), 2 (moderate), 3 (marked), and 4 (severe). All six categories were added together to garner a total pathology score with a maximum value of 24.

### Immunohistochemistry

Macrophages were detected with an anti-F4/80 antibody (1:50; BD Bioscience, San Jose, CA) and neutrophils were detected with anti-Ly6G antibody (1:50; BD Bioscience) in addition to their respective isotype controls. Following addition of HRP-conjugated secondary antibodies, slides were counter stained with H&E. F4/80 and Ly6G was quantified by digital images of every 5th field of view for the entire colon using Adobe Photoshop as previously described (26). NF-κB expression was detected by staining with a fluorescently labeled anti-p100/p52 antibody (1:100, Santa Cruz Biotech, TX) or anti-pNF-κB p65 (1:100, Santa Cruz Biotech). Nuclei were counterstained with DAPI. Relative ratios of p52/DAPI were determined using Image J.

### Semiquantitative Real-Time PCR

Total colonic, *in vitro* cell line, and *ex vivo* splenocyte RNA was isolated using a standard single-step isolation protocol (TRI-zol, Invitrogen, Carlsbad, CA). Complementary DNA was synthesized with the Avian Myeloblastosis Virus (AMV) Reverse Transcriptase kit (Promega Corporation, Madison, WI). Differences in gene expression were determined by Real-Time PCR (Quantstudio 3, Thermo Fisher, Foster City, CA). SYBR green was used in place of a labeled probe sequence for IL-22 and RegIIIγ. The relative amount of mRNA was determined using the comparative cycle threshold method (*C*_t_) as previously described (27, 28).

### Cell Culture

CECs, CMT-93 (ATCC CCL-223), and macrophages, RAW 264.7 (ATCC TIB-71), were cultured separately according to manufacturer's guidelines. CD11b^+^ splenocytes were isolated as previously described (29). Briefly, spleens were removed from male, C57BL/6 mice and macerated with glass slides. The resulting cell suspension was washed, filtered, and red blood cells were lysed. CD11b^+^ cells were isolated by use of Cd11b Microbeads (Miltenyi Biotec, Auburn, CA) according to the manufacturer's protocol and cultured in RPMI/10% FBS. Cells were treated with 50 μM sulfasalazine or 10 μM of PS-1145 dihydrochloride (Sigma Aldrich, St. Louis, MO) overnight (CMT-93 and RAW 264.7 cells) or 2 h (CD11b+ splenocytes). Cells were harvested for inflammatory and/or antimicrobial gene expression by RT-PCR. Spent supernatants were collected for TNF-α protein analysis via ELISA (R&D Systems, Minneapolis, MN).

### Flow Cytometry

Mesenteric lymph node cells were isolated and stained with FITC-conjugated CD11c (clone HL3) and APC-conjugated CD11b (clone M1/70) and FITC-conjugated CD3e (clone 145-2C11), PerCP-conjugated CD4 (clone RM4-5), and APC-conjugated CD8 (clone 53-6.7) (BD Pharmingen, San Jose, CA) as previously described (23). Samples were analyzed with the BD FACSCalibur dual-laser flow cytometer (BD Immunocytometry Systems, San Jose, CA) with CellQuest Pro. Cells were gated based on forward and side-scatter and CD11b, CD11c, CD3/CD4, or CD3/CD8 expression levels.

### DNA Extraction and Library Preparation

DNA was isolated from colonic tissue and contents using a QIAgen DNA Mini Kit (Qiagen, Valencia, CA) as previously described (30). DNA was quantified using the dsDNA Broad Range Assay Kit (Life Technologies, Carlsbad, CA), then sent to UNC Charlotte Department of Bioinformatics and Genomics for library preparation and sequencing. The V3–V4 hypervariable region of the 16S rRNA gene was the target for this study. Libraries were prepared with NexteraXT kit (Illumina, San Diego, CA) per manufacturer's instructions and equimolar samples pooled.

### Sequencing

Amplicon libraries were sequenced at UNC Charlotte using Illumina MiSeq resulting in 20,373,056 million paired-end reads (MiSeq Reagent Kit v3 600 cycle; Illumina). Forward and reverse reads were merged using Quantitative Insights into Microbial Ecology (QIIME) version 1.9.1 with an overlap length of 40 and 95% similarity in the overlap region. Trimming and filtering at Q20 resulted in 9,997,617 million reads. Trimmed and cleansed reads were loaded into QIIME. Closed reference OTU picking pipeline (at 97% similarity) along with green genes dataset version 13.8 was used to produce OTUs incorporating 77% of input reads. *De novo* OTU picking (at 97% similarity) was performed using AbuandantOTU+ version 0.92b. AbuandantOTU+ incorporated 98% of the input reads after removing chimeric and contaminant OTUs. Based on (31), a taxa was retained if it has 0.005% of the total count (31). Linear mixed effect model followed by ANOVA was conducted with Group, Infection, and Day as fixed effects and cage as a random effect (32). For separate time points and pairwise comparisons, the model includes only relevant terms. All *P*-values were FDR corrected. Alpha diversity was assessed using Chao1 and Shannon indexes using rarefied counts. Beta diversity was assessed using PCoAs designed from Bray-Curtis dissimilarity using log 10 normalized counts according to the following formula:

$log_{10}\langle \left(\frac{Texa\;raw\;count}{Number\;of\;sequence\;in\;sample}\times Average\;number\;of\;sequences\;per\;sample\right)\;+1\rangle$ (32, 33). The above mentioned approach was also applied to forward and reverse reads independently without merging to validate results from merged reads.

### Statistical Analysis

Changes in pathogen colonization, gene expression, and serum cytokine levels were analyzed by an analysis of variance (ANOVA) with mouse strain and days post-challenge as between or within subjects factors using IBM SPSS Statistics for Windows, Version 24.0 (SPSS, Chicago, IL). A mixed linear model where infection status, time point, tissue, and strain were considered fixed factors was used for sequencing analysis. *P*-values from sequencing data were FDR corrected. *Post-hoc* analysis for non-sequencing data comprised of two-tailed Student's *t*-test with Bonferroni correction applied.

## Results

### *Citrobacter rodentium* Colonization and Pathology Is Significantly Reduced in IKKβ-Deficient Mice

**Figure 1:**
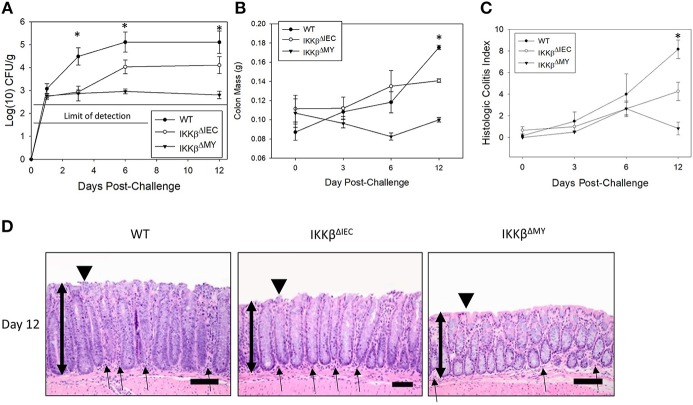
*Citrobacter rodentium* colonization and resulting infectious colitis are reduced in IKKβ-deficient mice. Wild type, IKKβ^ΔIEC^, and IKKβ^ΔMY^ mice were challenged with 3 × 10^8^ CFU of *C. rodentium*. **(A)** Colonic pathogen colonization was followed throughout the 12 days experiment. Stool samples were collected on Days 0, 3, 6, and 12 post-challenge and pathogen was enumerated via pour plate method. *Citrobacter rodentium* was significantly reduced in both IKKβ^ΔIEC^ and IKKβ^ΔMY^ throughout the 12 days experiment as compared to WT mice. *p < 0.001 IKKβ^ΔIEC^ or IKKβ^ΔMY^ vs. WT on Days 3, 6, and 12. **(B)** On Days 0, 3, 6, and 12 post-challenge, colons were removed, weighed, fixed in formalin, and embedded in paraffin. Wild type mice presented with a thickening of the colon on Day 12 post-challenge which was evident by enhanced mass, however there was a significant reduction in colon mass in both IKKβ^ΔIEC^ and IKKβ^ΔMY^ mice on Day 12 post-challenge. *p < 0.005 vs. WT. **(C)** Paraffin-embedded colons were sectioned and stained with hematoxylin and eosin in order to visualize and score the pathology present in each sample. There was a significant reduction in the total colitis index in IKKβ^ΔIEC^ and IKKβ^ΔMY^ mice as compared to WT. Colitis scores were also significantly reduced in IKKβ^ΔMY^ as compared to IKKβ^ΔIEC^ colons. *p < 0.0001 IKKβ^ΔIEC^ or IKKβ^ΔMY^ vs. WT. **(D)** Images are taken from representative H&E slides of a WT colon, IKKβ^ΔIEC^ colon, and IKKβ^ΔMY^ colon on Day 12 post-challenge. The degree of inflammatory cell infiltration in WT and IKKβ^ΔIEC^ is greater than IKKβ^ΔMY^, as noted by the subjectively increased number of cells. Single-headed arrows indicate the appearance of inflammatory cells within the base of the mucosa. Double-headed arrows indicate mucosal thickness, ▾ highlights the epithelial surface where there is noticeable epithelial tattering and erosion in WT and IKKβ^ΔIEC^ samples. *n* = 12 WT, *n* = 13 IKKβ^ΔIEC^, *n* = 6 IKKβ^ΔMY^. Data are the mean ± standard error.

*Citrobacter rodentium* colonization was significantly reduced in both IKKβ-deficient mice strains compared to wild type (WT) mice [*F*_(2, 61)_ = 4.475, *P* < 0.05; Figure 1A] with the most evident reduction occurring in IKKβ^ΔMY^ mice on Days 3, 6, and 12 post-challenge. Colon mass on Day 12 post-challenge is significantly increased in WT mice as compared to uninfected WT controls. This pathogen-induced increase was significantly reduced in both IKKβ^ΔMY^ and IKKβ^ΔIEC^ mice as compared to day-matched infected WT [*F*_(4, 61)_ = 6.36, *P* < 0.005; Figure 1B]. No overt colonic pathology or structural differences were observed in either IKKβ-deficient mice prior to bacterial challenge (Supplemental Figure 1). Total colitis index, as assessed by increases in colonic hyperplasia, dysplasia, inflammation, edema, and epithelial and crypt defects, was significantly reduced in each IKKβ-deficient strain on Day 12 post-challenge as compared to WT controls [*F*_(4, 40)_ = 3.3, *P* < 0.05; Figure 1C]. Colonic pathology centered on the mucosa and comprised of epithelial thickening with significant inflammatory cell infiltrates and epithelial damage, including focal ulcerations, on Day 12 post-challenge in WT mice (Figure 1D). IKKβ^ΔIEC^ colons also experienced a thickening of the mucosa along with enhanced inflammatory infiltrates; however, it was to a lesser extent as compared to WT colons. IKKβ^ΔMY^ colons had a relatively normal appearance. Because differences between WT and IKKβ deficient mice were most evident on Days 0, 3, and 12, these time points were further assessed.

### Colonic and Mesenteric Lymph Node Immune Populations Were Altered in IKKβ-Deficient Mice Following *C. rodentium* Challenge

**Figure 2:**
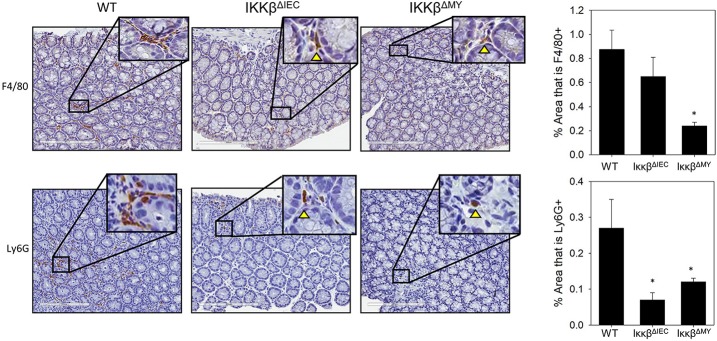
Colonic monocyte/macrophage and neutrophil accumulation is reduced in IKKβ-deficient mice. Colons were removed from mice on day 12 post-challenge and subsequently fixed in formalin, embedded in paraffin, then sectioned and stained with an antibody for the surface marker F4/80 in order to visualize macrophages or Ly6G to visualize neutrophils. The % area stained for either F4/80 or Ly6G was quantified from every 5th image of the entire length of the colon. Colonic monocyte/macrophage and neutrophil accumulation were reduced in IKKβ-deficient mice. Images are taken from slides representative of a WT, IKKβ^ΔIEC^, and IKKβ^ΔMY^. *p < 0.05 vs. WT *n* = 12 WT, *n* = 13 IKKβ^ΔIEC^, *n* = 6 IKKβ^ΔMY^. Data are the mean ± standard error.

**Figure 3:**
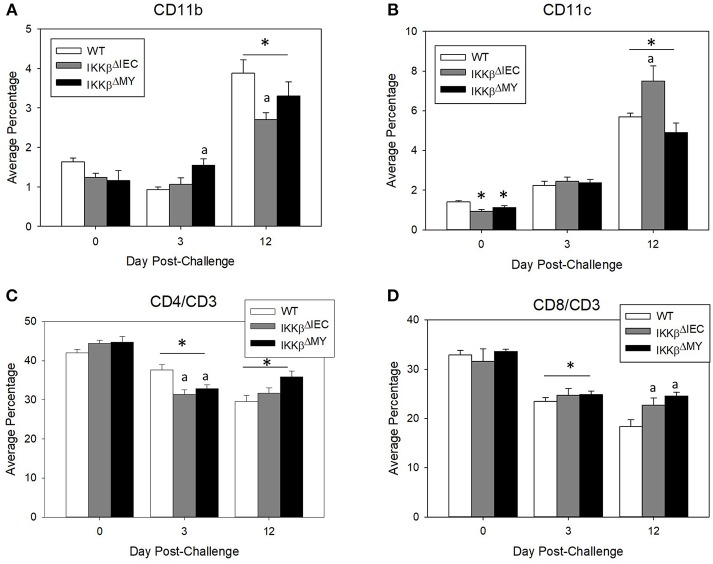
Mesenteric lymph node immune populations are altered in IKKβ-deficient mice following *C. rodentium* challenge. On Days 0, 3, and 12 post-challenge mesenteric lymph nodes were removed and processed for flow cytometry. **(A)** IKKβ^ΔMY^ mice experienced an increase in monocyte/macrophage accumulation on Day 3 post-challenge as compared Day 3 WT samples. There was also an increase in all groups on Day 12 post-challenge, however the increase was blunted in IKKβ-deficient mice. **(B)** Dendritic cells increased throughout the 12 days experiment as an effect of infection in all strains of mice, however IKKβ^ΔIEC^ mice experienced an increase in abundance over that of WT samples. **(C)** Helper T cell populations experienced a reduction in mesenteric lymph nodes between Day 3 and Day 12 post-challenge. **(D)** Cytotoxic T cells were increased in IKKβ-deficient mice as compared to WT on Day 12. *p < 0.01 vs. WT, *p* < 0.05 vs. Day matched WT. *n* = 12 WT, *n* = 13 IKKβ^ΔIEC^, *n* = 6 IKKβ^ΔMY^. Data are the mean ± standard error.

Naïve WT and IKKβ-deficient mice had similar levels of resident colonic monocytes/macrophages and neutrophils (data not shown). WT colons experienced an infection-dependent influx of monocytes/macrophages which was significantly reduced in IKKβ^ΔMY^ mice on Day 12 post-challenge [*F*_(2, 14)_ = 4.22, *p* < 0.05; Figure 2, top row]. Neutrophil accumulation was also reduced in IKKβ-deficient colons as evidenced by a significant reduction in IKKβ^ΔIEC^ mice on Day 12 post-challenge as compared to day-matched WT colons [*F*_(2, 12)_ = 5.27, *p* < 0.05; Figure 2, bottom row]. The immune profile of the mesenteric lymph node (MLN) was also altered following *C. rodentium* challenge. Monocyte/macrophage MLN accumulation was significantly altered in IKKβ-deficient mice on Days 3 and 12 post-challenge [*F*_(4, 40)_ = 5.375, *p* < 0.001; Figure 3A]. On Day 3 post-challenge there was a significant increase of CD11b^+^ mononuclear cells in IKKβ^ΔMY^ mice as compared to day-matched WT. Additionally, on Day 12 post-challenge, IKKβ^ΔIEC^ mice experienced a significant reduction of CD11b^+^ mononuclear cell accumulation compared to day-matched WT mice. Dendritic cells were also significantly increased in MLN following *C. rodentium* infection [*F*_(4, 45)_ = 7.003, *p* < 0.001; Figure 3B]. This effect was regardless of strain, however there was a significant increase in CD11c^+^ dendritic cells in IKKβ^ΔIEC^ mice on Day 12 post-challenge as compared to day-matched WT mice. The prevalence of both CD4^+^ and CD8^+^ T cells were altered in IKKβ-deficient mice as compared to WT mice. For instance there was a significant reduction of CD3^+^CD4^+^ T cells in IKKβ-deficient MLN on Day 3 post-challenge compared to day-matched WT [*F*_(4, 40)_ = 4.961, *p* < 0.005; Figure 3C]. A significant reduction of MLN CD3^+^CD4^+^ T cells was observed on Day 12 post-challenge in WT mice compared to Day 3 post-challenge WT mice. This reduction was not observed in IKKβ-deficient mice. Furthermore, CD3^+^CD8^+^ T cell levels were altered in IKKβ-deficient MLNs as compared to WT MLNs as evidenced by an overall increase in CD3^+^CD8^+^ percentage within IKKβ-deficient mice as compared to WT mice on Days 3 and 12 [*F*_(2, 41)_ = 49.11, *p* < 0.001, Figure 3D].

### Colonic Gene Expression Is Altered in IKKβ-Deficient Mice Before and During *C. rodentium* Challenge

**Figure 4:**
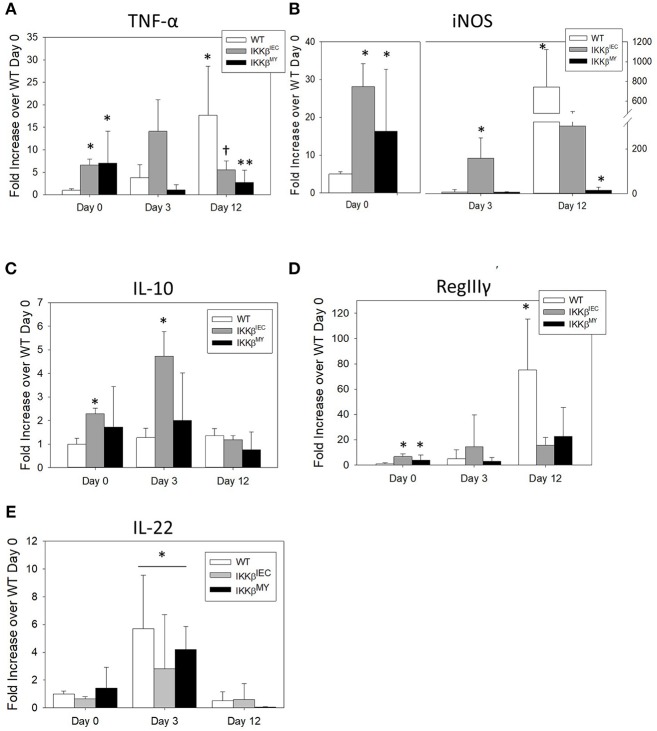
Pathogen-induced colonic mRNA expression is altered in IKKβ-deficient mice. Wild type, IKKβ^ΔIEC^, and IKKβ^ΔMY^ mice were challenged with 3 × 10^8^ CFU of *C. rodentium*. On Days 0, 3, and 12 post-challenge, colons were removed and processed in order to quantify mRNA expression by Real Time PCR. **(A)** TNF-α was significantly increased in both IKKβ^ΔIEC^ and IKKβ^ΔMY^ on Day 0 as compared with WT colons. By Day 12 post-challenge there were decreases in TNF-α of both IKKβ^ΔIEC^ and IKKβ^ΔMY^ as compared to Day 12 WT samples. *p < 0.05 vs. WT, ^†^ < 0.06 IKKβ^ΔIEC^ vs. Day-matched WT. **(B)** iNOS expression was significantly increased on Day 0 in IKKβ^ΔIEC^ and IKKβ^ΔMY^ and on Day 3 in IKKβ^ΔIEC^ as compared to WT On Day 12 post-challenge the increase in WT colons was significantly reduced in IKKβ^ΔMY^ colons. *p < 0.05. **(C)** There was a significant increase in IL-10 gene expression in IKKβ^ΔIEC^ colons on Days 0 and 3 post-challenge as compared to Day-matched WT colons. *p < 0.001 IKKβ^ΔIEC^ vs. WT. **(D)** RegIIIγ expression was significantly increased in colons from both IKKβ^ΔIEC^ and IKKβ^ΔMY^ mice as compared with colons from WT controls on Day 0. Colons from WT mice experienced a significant increase on Day 12 post-challenge. *p < 0.001 IKKβ^ΔIEC^ or IKKβ^ΔMY^ vs. WT. **(E)** IL-22 expression was significantly increased on Day 3 post-challenge as compared to Day 0 and Day 12 post-challenge. *p < 0.01 Day 3 vs. Day 0 and Day 12. *n* = 12 WT, *n* = 13 IKKβ^ΔIEC^, *n* = 6 IKKβ^ΔMY^. Data are the mean ± standard error.

On Day 12 post-challenge there was a significant increase in colonic tumor necrosis factor (TNF)-α expression in infected WT mice as evidenced by a 20-fold increase over non-infected, i.e., D0, WT colons. This effect was not observed in IKKβ-deficient colons [*F*_(4, 48)_ = 4.9, *p* < 0.005; Figure 4A]. The enzyme inducible nitric oxide synthase (iNOS) expression was also significantly increased on Day 12 post-challenge in WT mice [*F*_(4, 52)_ = 9.5, *P* < 0.001; Figure 4B]. On Day 12 post-challenge, there was a non-significant reduction in colonic iNOS expression in IKKβ^ΔIEC^ mice and a significant reduction in IKKβ^ΔMY^ mice compared to day-matched WT mice (*p* < 0.05; Figure 4C). Interestingly, prior to pathogen challenge there was a significant increase in basal levels of colonic TNF-α and iNOS expression in both uninfected IKKβ^ΔIEC^ and IKKβ^ΔMY^ mice compared to non-infected WT with a 10-fold increase in TNF-α and a 20-fold increase in iNOS in uninfected knockouts compared to uninfected WT mice (Figures 4A,B). Colonic gene expression of the anti-inflammatory cytokine interleukin (IL)-10 was unchanged throughout the 12 day experiment in WT and IKKβ^ΔMY^ mice (Figure 4C). Uninfected, IKKβ^ΔIEC^ mice experienced a 2 fold increase of IL-10 expression and a 4-fold increase on Day 3 post-challenge as compared to non-infected WT mice [*F*_(4, 48)_ = 4.0, *p* < 0.01; Figure 4C]. The antimicrobial peptide RegIIIγ experienced a 75-fold increase in WT colons on Day 12 post-challenge as compared to non-infected WT colons [*F*_(4, 48)_ = 3.3, *p* < 0.05; Figure 4D]. This infection-induced increase of RegIIIγ was not observed in either IKKβ^ΔMY^ or IKKβ^ΔIEC^ mice. However, similar to TNF-α and iNOS expression, there were significant increases of colonic RegIIIγ in non-infected IKKβ^ΔMY^ or IKKβ^ΔIEC^ mice as compared to non-infected WT mice. The cytokine IL-22 was significantly increased following *C. rodentium* challenge as observed by an overall increase on Day 3 post-challenge in all strains of mice [*F*_(2, 35)_ = 11.05, *p* < 0.001; Figure 4E] as compared to non-infected mice and Day 12 post-challenge.

### Baseline and Pathogen-Induced Colonic NF-κB Is Altered in IKKβ-Deficient Mice

**Figure 5:**
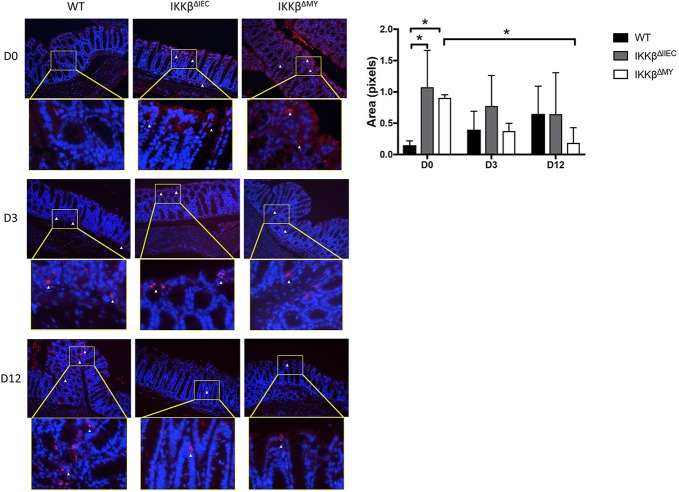
Colons from IKKβ^ΔIEC^ and IKKβ^ΔMY^ exhibit enhanced p52 expression. Colons were removed from mice on Days 0, 3, and 12 post-challenge and subsequently fixed in formalin, embedded in paraffin, then sectioned and stained for p52 (red) and counterstained with DAPI (blue). Colons from naïve IKKβ-deficient mice had significant differences in p52 staining compared to WT mice. This was most evident on Day 0, where IKKβ^ΔIEC^ and IKKβ^ΔMY^ had significantly higher p52 staining than did WT mice *p < 0.05. p52 staining was also significantly lower in IKKβ^ΔMY^ mice on Day 12 vs. Day 0 of *C. rodentium* *p < 0.05. Relative ratios of p52 staining/DAPI were quantified with Image J. Images are taken from slides representative of a WT, IKKβ^ΔIEC^, and IKKβ^ΔMY^. *n* = 3 WT, *n* = 4 IKKβ^ΔIEC^, *n* = 3 IKKβ^ΔMY^ for each day post-challenge.

Consistent with changes in cytokines, colonic p52 expression, an NF-κB subunit involved in the alternative pathway, was significantly different in IKKβ^ΔIEC^ and IKKβ^ΔMY^ mice compared to WT mice. On Day 0, i.e., prior to *C. rodentium* exposure, there was evidence of higher p52 expression in colons from both IKKβ^ΔIEC^ and IKKβ^ΔMY^ mice compared to uninfected WT mice, in which p52 expression was low to undetectable (*p* < 0.05; Figure 5). Although *C. rodentium* challenge led to increases in p52 expression in WT mice, p52 did not increase in IKKβ^ΔIEC^ challenged with *C. rodentium*, and was significantly decreased in IKKβ^ΔMY^ mice by Day 12 post-challenge (*p* < 0.05; Figure 5). Colonic p65 was also activated as assessed by phosphorylated p65 (p-p65) in mice on Days 3 and 12 post challenge (Supplemental Figure 2). Phosphorylation of p65 was evident in CECs of IKKβ^ΔIEC^ mice on Day 3 and 12 post-challenge, however it appears to be confined to the cytoplasm, whereas colonic p-p65 appeared to be localized to the nuclei of epithelial cells in IKKβ^ΔIMY^ colons, but not the lamina propria.

### Pharmacologic Blockade of Classical NF-κB Leads to Altered Cytokine Expression in Colonic Epithelial Cells and Macrophages

Sulfasalazine was used to block classical NF-κB in CMT-93 cells, RAW 264.7 cells, and *ex vivo* CD11b^+^ splenocytes (34, 35). RT-PCR revealed increased epithelial cell iNOS and RegIIIβ gene expression in sulfasalazine-treated CMT-93 cells (*p* < 0.05, Supplemental Figure 3A). Sulfasalazine treatment also increased TNF-α gene expression in RAW 264.7 macrophages (*p* < 0.05, Supplemental Figure 3B). Pharmacologic blockade of classical NF-κB in primary CD11b^+^ splenocytes also lead to a significant increase in TNF-α gene expression (*p* < 0.05, Supplemental Figure 3C). A separate set of experiments were performed in which CMT-93 and RAW 264.7 cells were treated overnight with PS-1145 dihydrochloride, an IKKβ-specific inhibitor. Similar to sulfasalazine treatment, CMT-93 cells experienced no change in TNF-α expression, a non-significant increase in iNOS expression and a significant increase in RegIIIβ expression following overnight PS-1145 treatment (*p* < 0.05, Supplemental Figure 3D) when compared to non-treated cells. PS-1145 treatment also lead to a significant increase in RAW 267.4 TNF-α gene expression (*p* < 0.01, Supplemental Figure 3E) and protein levels (*p* < 0.05, Supplemental Figure 3F) compared to untreated cells. TNF-α protein levels were undetectable in CMT-93 supernatants (data not shown).

### Colonic Bacterial Diversity Is Different in IKKβ-Deficient Mice Prior to and During *C. rodentium* Challenge

**Figure 6:**
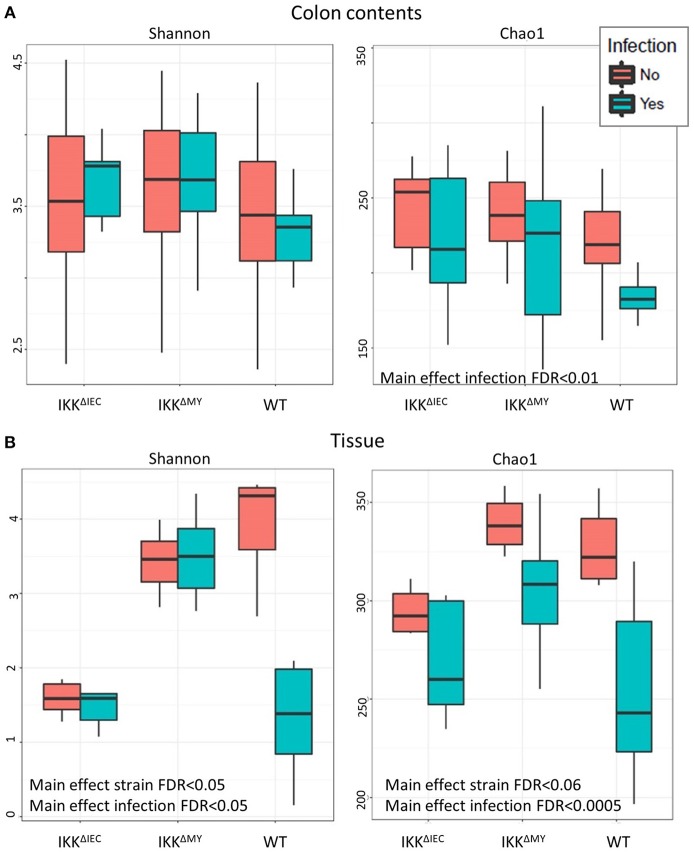
The colonic microbial alpha diversity is affected by IKKβ deficiency and *C. rodentium* infection. **(A)** There is a main effect of infection in colonic contents as assessed by Chao1 as indicated by an overall reduction of diversity due to *C. rodentium* infection. FDR < 0.01. **(B)** Tissue-associated populations are also distinctly different due to both strain and infection in each Shannon and Chao1. Alpha diversity was reduced in IKKβ^ΔIEC^ and IKKβ^ΔMY^ as compared to WT samples as assessed by the Shannon Diversity Index. Alterations due to infection resulted from a significant reduction of diversity in WT samples. Alpha diversity as assessed by Chao1 was also altered due to both strain and *C. rodentium* infection as evidenced by a reduction of diversity following infection.

There were no differences in the Shannon Diversity Index (SDI) in either non-infected or infected colonic contents (Figure 6A). However, when Chao1 was used as a measure of alpha diversity, there was a main effect with regard to infection, likely due to reduced alpha diversity in WT colonic contents following bacterial infection (FDR < 0.01; Figure 6A). Tissue-associated alpha diversity was also significantly different based on strain and infection status. SDI demonstrated a main effect with regard to strain with the most evident change being lower alpha diversity in IKKβ^ΔIEC^ colonic tissue compared to WT and IKKβ^ΔMY^ mice (FDR < 0.05; Figure 6B). Pathogen challenge also caused a significant change in alpha diversity as assessed by SDI, which was mainly due to reduced diversity within WT tissue samples following *C. rodentium* challenge (FDR < 0.05; Figure 6B). Colonic tissue alpha diversity as assessed by Chao1 mirrored that of SDI in that there was a main effect of strain (FDR < 0.06) and infection (FDR < 0.0005; Figure 6B).

**Figure 7:**
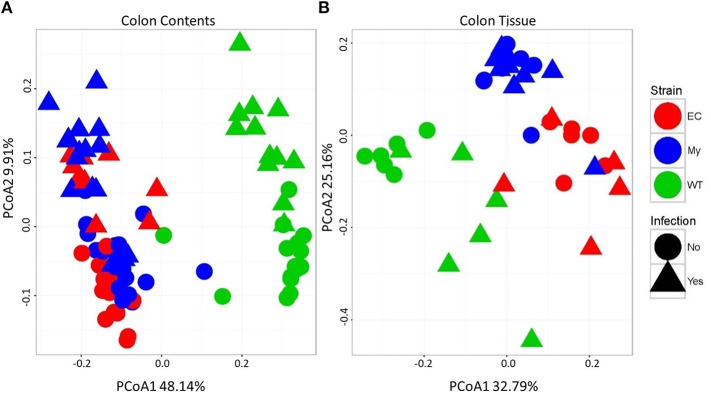
Colonic microbial beta diversity is distinctive in animals deficient in classical NF-κB signaling or infected with *C. rodentium* compared to WT or uninfected animals. Principal coordinated analysis (PCoA) using Bray-Curtis dissimilarity using log_10_ normalized counts to demonstrate strain and infection-induced differences in **(A)** colonic contents and **(B)** colonic tissue.

Differences in microbial communities, as determined by beta diversity, were visualized using Principal Coordinates Analysis (PCoA) built from Bray-Curtis dissimilarity using log_10_ normalized counts. Prior to pathogen challenge, there were significant differences between the microbial communities present within the colonic contents of WT mice vs. IKKβ-deficient strains (FDR <1.0 × 10^−10^; Figure 7A, circles). Microbial populations from IKKβ^ΔIEC^ and IKKβ^ΔMY^ colonic contents were also distinct from each other (PCoA2; FDR < 0.00005), however this difference was not as great as the difference between WT and IKKβ-deficient populations. Infected mice had a shift in contents-associated microbial communities in each mouse strain (PCoA1; FDR < 0.00001; Figure 7A, triangles). Tissue-associated microbial communities were also distinct with regard to strain prior to bacterial challenge (PCoA1; FDR < 0.0001; Figure 7B, circles). *C. rodentium*-challenge caused shifts of tissue-associated microbial communities (PCoA2; FDR < 0.05; Figure 7B, triangles).

### Tissue-Associated and Contents-Associated Microbial Taxa Are Significantly Altered in IKKβ-Deficient Mice Prior to and During *C. rodentium* Challenge

**Figure 8:**
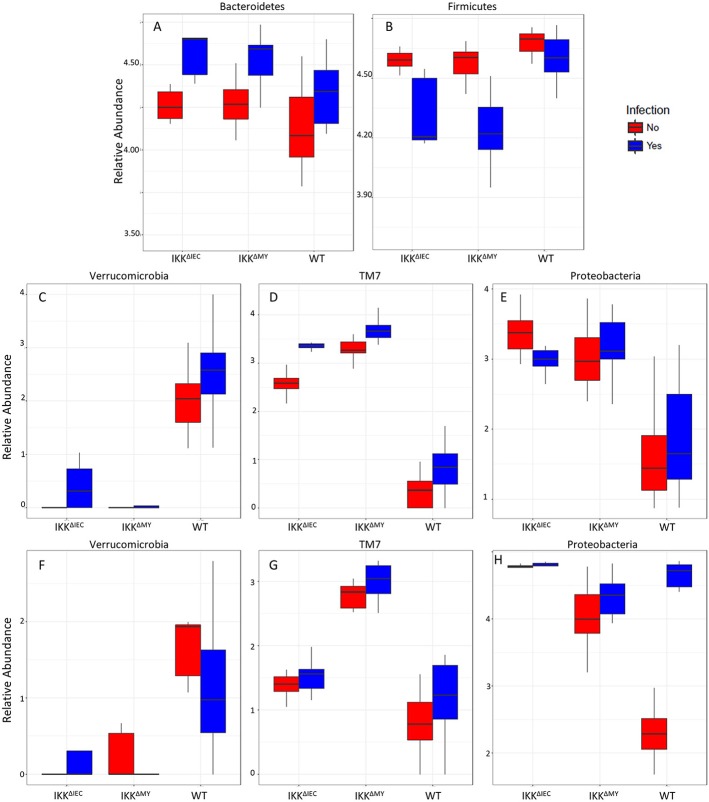
Bacterial phyla are affected by *C. rodentium* infection in WT and IKKβ-deficient mice. Phyla relative abundances of colonic contents for **(A)** Bacteroidetes, **(B)** Firmicutes, **(C)** Verrucomicrobia, **(D)** TM7, **(E)** Proteobacteria, and tissue-associated phyla relative abundances for **(F)** Verrucomicrobia, **(G)** TM7, and **(H)** Proteobacteria.

Bacteroidetes populations within colonic contents were higher in IKKβ-deficient strains compared to WT mice as indicated by a main strain effect in both non-infected and infected mice (FDR < 0.01; Figure 8A). Infection also significantly increased colonic content Bacteroidetes levels as evidenced by main effect of infection (FDR < 0.0005; Figure 8A). Firmicutes populations were also different as evidenced by a main effect of strain (FDR < 0.005; Figure 8B) in addition to pathogenic infection (FDR < 0.00001; Figure 8B). Overall Firmicutes were lower in IKKβ-deficient colonic contents as compared to WT contents. Infection caused a reduction of Firmicutes in IKKβ-deficient mice, but not WT (Figure 8B). Verrucomicrobia, TM7, and Proteobacteria were all significantly different in the colonic contents (Figures 8C–E) and tissue (Figures 8F–H) of IKKβ-deficient mice prior to and during *C. rodentium* infection. Verrucomicrobia populations were significantly lower (and in some animals undetectable) in IKKβ^ΔIEC^ and IKKβ^ΔMY^ colonic contents compared to WT mice prior to pathogen challenge (FDR < 0.00001; Figure 8C). Conversely, there were significantly higher levels of TM7 populations in IKKβ-deficient colonic contents as compared to WT mice prior to and during bacterial challenge (FDR < 0.000000005; Figure 8D). Proteobacteria levels were significantly higher in IKKβ-deficient mice in both non-infected and infected contents as compared to WT colonic contents (FDR < 0.000005; Figure 8E). Tissue-associated bacterial taxa were also distinct based on the genetic status of the mouse. Similar to colonic contents, tissue-associated Verrucomicrobia were significantly lower in IKKβ-deficient mice in comparison to WT mice (FDR < 0.01; Figure 8F). Tissue-associated TM7 were significantly higher in tissue-associated populations in IKKβ^ΔMY^ mice (FDR < 0.00001; Figure 8G). Neither tissue-associated Verrucomicrobia nor TM7 were different following *C. rodentium* challenge. Tissue-associated Proteobacteria in IKKβ-deficient mice were significantly higher as compared to WT tissues (FDR < 0.01; Figure 8H). Pathogen challenge was associated with a significant increase in tissue-associated Proteobacteria that was most evident in WT colon samples (FDR < 0.005; Figure 8H).

**Figure 9:**
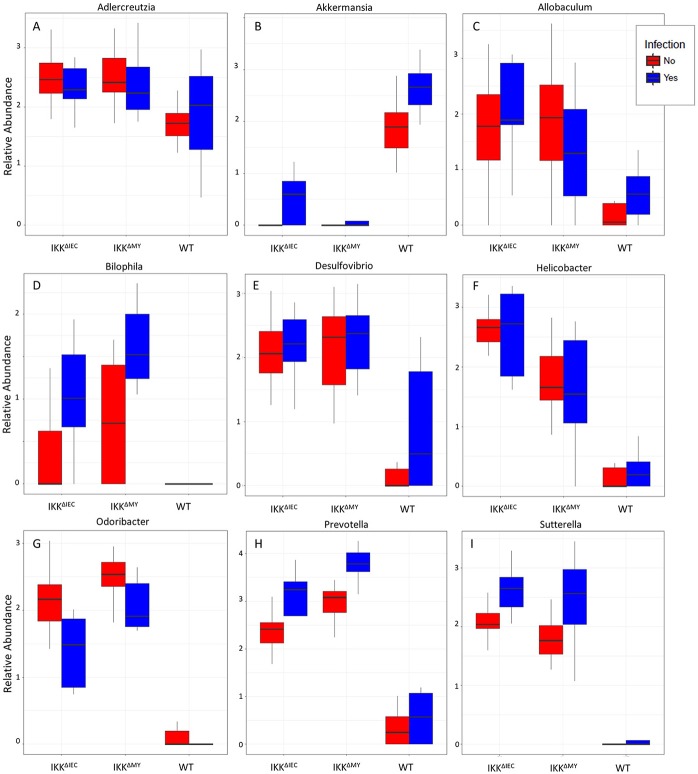
Differences in bacterial genera are evident in IKKβ-deficient mice prior to and during *C. rodentium* challenge. Uninfected and infected colonic contents genera relative abundances for **(A)** *Adlercreutzia*, **(B)** *Akkermansia*, **(C)** *Allobaculum*, **(D)** *Bilophila*, **(E)** *Desulfovibrio*, **(F)** *Helicobacter*, **(G)** *Odoribacter*, **(H)** *Prevotella*, and **(I)** *Sutterella*.

**Figure 10:**
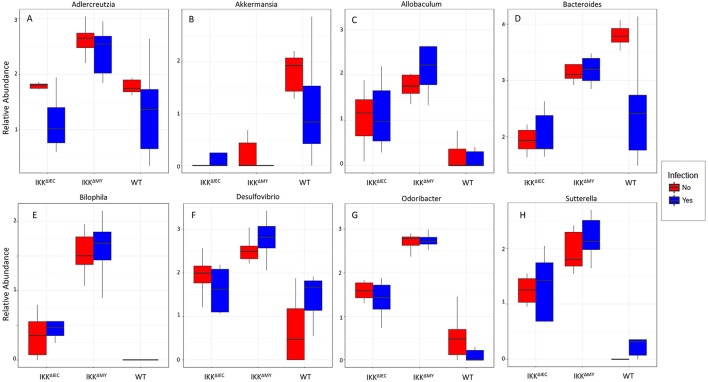
Tissue-associate bacterial genera are different in *C. rodentium*-infected and in IKKβ-deficient mice. Colonic tissue-associated genera relative abundance for **(A)** *Adlercreutzia*, **(B)** *Akkermansia*, **(C)** *Allobaculum*, **(D)** *Bacteroides*, **(E)** *Bilophila*, **(F)** *Desulfovibrio*, **(G)** *Odoribacter*, and **(H)** *Sutterella*.

Lower bacterial taxa were also different in IKKβ-deficient mice prior to and during *C. rodentium* challenge. *Adlercreutzia* (FDR < 0.01; Figure 9A), *Allobaculum* (FDR < 0.005; Figure 9C), *Bilophila* (FDR < 0.0001; Figure 9D), *Desulfovibrio* (FDR < 0.00001; Figure 9E), *Helicobacter* (FDR < 0.000001; Figure 9F), *Odoribacter* (FDR < 0.0000001; Figure 9G), *Prevotella* (FDR < 0.0000001; Figure 9H), and *Sutterella* (FDR < 0.0000001; Figure 9I) all were significantly higher in non-infected colonic contents from IKKβ-deficient mice as compared to WT mice. Conversely, *Akkermansia* were significantly lower in stool from IKKβ-deficient mice as compared to WT (FDR < 0.0000005; Figure 9B). Infection did not alter *Adlercreutzia, Allobaculum, Desulfovibrio*, and *Helicobacter* prevalence in colonic contents of either IKKβ-deficient or WT mice. There were infection-induced increases in *Bilophila* (FDR < 0.005; Figure 9D), *Prevotella* (FDR < 0.01; Figure 9H), and *Sutterella* (FDR < 0.01; Figure 9I), however the increases only occurred in IKKβ^ΔIEC^ and IKKβ^ΔMY^ mice. *Odoribacter* was reduced by *C. rodentium* challenge in IKKβ^ΔIEC^ and IKKβ^ΔMY^ mice and to undetectable levels in colonic contents from WT mice (FDR < 0.0005; Figure 9G). Tissue-associated bacterial populations were also distinct between IKKβ-deficient and WT mice prior to and during *C. rodentium* infection. Tissue-associated *Allobaculum* (FDR < 0.0001; Figure 10C), *Bilophila* (FDR < 0.000001; Figure 10E), *Desulfovibrio* (FDR < 0.001; Figure 10F), and *Sutterella* (FDR < 0.00001; Figure 10H) all were higher in uninfected IKKβ^ΔIEC^ and IKKβ^ΔMY^ colon samples compared to uninfected WT tissue. Tissue-associated *Akkermansia* (FDR < 0.01; Figure 10B) and *Bacteroides* (FDR < 0.01; Figure 10D) were lower in uninfected IKKβ^ΔIEC^ and IKKβ^ΔMY^ tissue samples as compared to uninfected WT tissue. *Adlercreutzia* (FDR < 0.005; Figure 10A) and *Odoribacter* (FDR < 0.00005; Figure 10G) were increased in uninfected tissue-associated IKKβ^ΔMY^ samples as compared to WT samples. Infection was not associated with differences in populations of *Akkermansia, Allobaculum, Bilophila, Desulfovibrio, Odoribacter*, or *Sutterella*. However, *Adlercreutzia* (FDR < 0.05; Figure 10A) was lower in *C. rodentium*-challenged mice.

## Discussion

Results of this study highlight the importance of CECs and myeloid-derived cells for *C. rodentium* challenge. Mice that are unable to activate classical NF-κB in intestinal epithelial cells, i.e., IKKβ^ΔIEC^ mice, have a reduction in *C. rodentium*-induced colon mass, colonic histopathology, and pathogen-induced colonic inflammatory mediator gene expression compared to WT controls. Interestingly, a further reduction in infection-induced colon mass, histopathology, and colonic gene expression is observed in mice with defective NF-κB signaling within myeloid-derived cells, i.e., IKKβ^ΔMY^ mice. While these results are striking, a confound presents as there is significantly lower colonic pathogen colonization in both strains of IKKβ-deficient compared to WT mice. *C. rodentium*-induced colonic pathology parallels pathogenic burden, therefore it is possible the reductions in colonic pathology are simply due to pathogen burden reduction (3). While this may be likely, it must be noted that all mice were inoculated with 3 × 10^8^ CFU *C. rodentium*; however, the colonization rates vary drastically between different strains. Pathogen burden was heaviest within WT mice with maximum colonization reaching over 10-fold over IKKβ^ΔIEC^ mice and 100-fold over IKKβ^ΔMY^ mice. This coincided with 82% of WT mice becoming colonized, 70% of IKKβ^ΔIEC^ mice were colonized, and 50% of IKKβ^ΔMY^ mice were colonized. To decipher why IKKβ^ΔIEC^ and IKKβ^ΔMY^ mice were protected from *C. rodentium* colonization and resulting histopathology, we examined colonic gene expression and bacterial profiles prior to pathogen challenge.

Recent evidence highlights the importance of CEC-derived NF-κB activation during intestinal homeostasis and inflammation. The full blockade of epithelial-derived NF-κB activation via deletion of epithelial-specific IKKγ led to severe spontaneous colitis prior to weaning (36). Inflammation was confined to the colon and characterized by enhanced immune infiltration and proinflammatory cytokine and chemokine production. Further investigation demonstrated that epithelial-derived NF-κB is necessary to maintain barrier homeostasis by controlling the bacterial populations that reside within the colon. Giacomin et al. demonstrated that alternative NF-κB activation via IKKα conferred protection to mice during *C. rodentium* challenge (37). IKKα-induced activation leads p52/RelB subunit activation. The beneficial effects of IKKα-derived NF-κB activation during *C. rodentium*-challenge were attributed to enhanced IL-22 induced antimicrobial peptides RegIIIγ and RegIIIβ. Interestingly, colons from both IKKβ^ΔIEC^ and IKKβ^ΔMY^ mice experienced enhanced basal colonic TNF-α, iNOS, and RegIIIγ gene expression, as well as increased p52 expression, prior to pathogen challenge as compared to uninfected WT colons, even though IL-22 expression was not enhanced. Although there was no evidence of enhanced histopathology in the absence of pathogen challenge in either IKKβ-deficient strain, the increases in baseline TNF-α, iNOS, and RegIIIγ suggested that these inflammatory mediators can be increased upon inhibition of classical NF-κB. This was confirmed via *in vitro* testing using specific, classical NF-κB pathway inhibitors. The increased inflammatory mediators at baseline (i.e., prior to pathogen challenge) led us to determine whether bacterial communities from IKKβ^ΔIEC^ and IKKβ^ΔMY^ mice were different than WT mice.

There is gaining evidence that suggests mucosal pathogen colonization must be met with a certain degree of inflammation (38). One theory is that inflammation leads to resident microbiome shifts which pathogens take advantage of in order for colonization. Evidence to support this theory was revealed in a study performed by Lupp et al., which demonstrated *C. rodentium*-challenge significantly alters the microbiota, the kinetics of which paralleled pathogen-induced inflammatory response; as the inflammatory response to pathogen increased, the colonic bacterial diversity decreased, which may have allowed for the increased *C. rodentium* colonization (38). Within our model, we did not observe significant differences in alpha diversity in non-infected colonic contents, however there was an overall strain effect in both Shannon and Chao1 alpha diversity in tissue-associated bacterial populations. Infection did cause overall reductions of alpha diversity, however the most striking reductions were in populations from WT mice. The increased susceptibility of WT mice to *C. rodentium* is consistent with studies showing that lower α diversity is associated with increased susceptibility to enteric infection. Beta diversity analysis also indicated significant differences in microbial populations between naïve WT, IKKβ^ΔIEC^, and IKKβ^ΔMY^ mice with tissue-associated or stool-associated populations being most dissimilar between WT and IKKβ-deficient mice. *C. rodentium* challenge did cause beta diversity shifts in all stool-associated bacteria from all strains of mice, however beta diversity alterations were most evident in WT tissue-associated populations.

There are multiple avenues through which microorganisms can aide their host to protect against foreign invaders, including antimicrobial peptide induction, shaping the immune system, and nutrient competition, thus blooms in different taxa can confer either resistance or susceptibility to infection. Historically, *C. rodentium* resistance has been correlated with increases in Bacteroidetes and reductions in Firmicutes (39, 40). However, differences in Bacteroidetes and Firmicutes do not appear to account for differences in susceptibility to *C. rodentium* in this study, since colonic contents from IKKβ^ΔIEC^ and IKKβ^ΔMY^ mice had higher Bacteroidetes and lower Firmicutes compared to WT mice. These findings in the colon are different than findings in the small intestine where the Bacteroidetes to Firmicutes ratio is similar in WT and IKKβ^ΔIEC^ and IKKβ^ΔMY^ mice (18). Although other phyla were different in the colons of IKKβ-deficient mice compared to WT mice, differences in Proteobacteria were associated with resistance to C. rodentium colonization. Prior to pathogen challenge, WT mice had a lower abundance of colonic mucosa-associated Proteobacteria than did IKKβ-deficient mice. This finding is noteworthy within the context of *C. rodentium* challenge, since *C. rodentium* is a member of the Proteobacteria phylum. Kamada et al. have shown using mice monoassociated with a common member of the Proteobacteria phylum, e.g., *E. coli*, that Proteobacteria can outcompete *C. rodentium* in the colon and thus confer protection (8). Similar findings were not evident in mice monoassociated with common members of other bacterial phyla, such as Bacteroidetes, e.g., *Bacteroides thetaiotaomicron* or *B. vulgatus* (8). In the current study, WT mice had lower Proteobacteria prior to infectious challenge, but after challenge, WT mice had higher Proteobacteria associated with the colonic mucosa due to the increased colonization with *C. rodentium*.

In the colon, commensal bacteria generally do not come into contact with CECs due to the thick mucus layer (41, 42). Members of the *Akkermansia* and *Allobaculum* genera were different in a pattern that predicts IKKβ-deficient mice have a more stable mucus layer as compared to WT mice. Numerous *Akkermansia* species have been indicated in mucus degradation and is significantly reduced in stool-associated and tissue-associated IKKβ-deficient mice as compared to WT mice. In contrast, *Allobaculum* members can promote mucus production and are significantly increased in stool-associated and tissue-associated IKKβ-deficient samples compared to WT samples. Proteobacteria members *Bilophila* and *Desulfovibrio*, and the Bacteroidetes member *Prevotella* are all significantly higher in the colonic contents and tissue of IKKβ-deficient mice over WT mice. Members of the aforementioned genera are able to reduce sulfur compounds produced during mucus degradation to dihydrogen sulfide (43). Currently the role of colonic hydrogen sulfide as it pertains to inflammation is controversial, however studies have indicated that blocking hydrogen sulfide synthesis exacerbated experimental colitis and endogenous hydrogen sulfide had anti-inflammatory effects on experimental colitis that was attributed to its ability to reduce colonic neutrophil recruitment (44–46). *Odoribacter*, a member of the Bacteroidetes, is associated with gastrointestinal health due to its ability to produce short chain fatty acids (47), such as butyrate, which CECs use as a nutrient source.

Further studies are needed to elucidate the meaning of alternative NF-κB-induced changes to gastrointestinal immunity to *C. rodentium*, however the results of this study highlight the deleterious effect that pathogen-induced classical NF-κB signaling within epithelial and myeloid-derived cells can have during gastrointestinal infection. The absence of the IKKβ subunit was associated with increased activation of the alternative NF-κB pathway in the absence of pathogen challenge which could be due to overcompensation by the IKKα subunit. Prior to pathogen challenge, enhanced alternative NF-κB activation, as indicated by increased p52, was associated with significant alterations in the colonic microenvironment, including increased TNF-α, iNOS, and RegIIIγ in the IKKβ-deficient mice. Cell culture studies also showed that treatment with a pharmacological inhibitor of classical NF-κB in *in vitro* CEC and macrophages and primary CD11b^+^ splenocytes lead to increases in TNF-α, iNOS, and RegIIIβ gene expression. Thus, it is possible that enhanced colonic cytokines and antimicrobial peptides could lead to shifts in bacterial communities, such as increased levels of the mucus production enhancer *Allobaculum* and reductions of the mucin-degrader *Akkermansia* in IKKβ-deficient mice. Additionally, increased levels of resident Proteobacteria are protective against *C. rodentium* and likely rendered IKKβ-deficient mice resistant to *C. rodentium* challenge. This increased resistance of IKKβ-deficient mice to *C. rodentium* challenge prevented the post-challenge increase in pathogen levels, pathogen-induced histologic colitis, alternative NF-κB activation, and inflammatory cytokines observed in WT mice.

## Ethics Statement

This study was carried out in accordance with the recommendations of The Ohio State University's Animal Care and Use Committee. The protocol was approved by the Animal Care and Use Committee.

## Author Contributions

AM and MB designed the study, developed methodology, collected and analyzed data, and wrote the manuscript. EK, JA, CL, and PB collected and analyzed data. NP analyzed data. CM and RG developed methodology and collected data. All authors edited and provided critical analysis of manuscript.

### Conflict of Interest Statement

The authors declare that the research was conducted in the absence of any commercial or financial relationships that could be construed as a potential conflict of interest.

## Supplementary Material

Supplemental Figure 1 Non-infected mice do not exhibit enhanced colonic histopathology. Paraffin-embedded colons were sectioned and stained with hematoxylin and eosin in order to visualize and score the pathology present in each sample. Representative images from non-infected mice are presented. *n* = 3/group.

Supplemental Figure 2 Classical p65 expression is increased during *C. rodentium* challenge. Paraffin-embedded colons were sectioned and stained for phosphorylated p65 (red) and counterstained with DAPI (blue). *n* = 3/group.

Supplemental Figure 3 Sulfasalazine and PS-1145 leads to altered gene expression in colonic epithelial cells and macrophages. **(A)** CMT-93 colonic epithelial cells. **(B)** RAW 264.7 macrophages and **(C)** CD11b^+^ *ex vivo* cultured splenocytes were inoculated with sulfasalazine (50 μm) overnight (CMT-93 and RAW 264.7) or 2 h (CD11b^+^). **(A)** Sulfasalazine enhanced baseline inflammatory (iNOS) and antimicrobial gene expression (RegIIIβ) in cultured CMT-93 cells ^*^*p* < 0.05. Sulfasalazine treatment also increased TNF-α gene expression in **(B)** RAW 264.7 macrophages (^*^*p* < 0.05) and **(C)** CD11b^+^ monocytes (^*^*p* < 0.05). **(D)** CMT-93 colonic epithelial cells. **(E,F)** RAW 264.7 macrophages were treated with PS-1145 (10 μm) overnight. **(D)** PS-1145 significantly increased baseline RegIIIβ gene expression in CMT-93 cells ^*^*p* < 0.05. **(E)** PS-1145 treatment significantly increased TNF-α expression in RAW 264.7 cells ^*^*p* < 0.01. **(F)** TNF-α protein levels were also significantly increased by PS-1145 treatment in RAW 264.7 macrophages ^*^*p* = 0.05.
