# Supplementary figures and images for: Mice Deficient in Epithelial or Myeloid Cell Iκκβ Have Distinct Colonic Microbiomes and Increased Resistance to Citrobacter rodentium Infection
Source: Front Immunol. 2019 Sep 10;10:2062. doi: 10.3389/fimmu.2019.02062 (PMC6746829; doi:10.3389/fimmu.2019.02062)

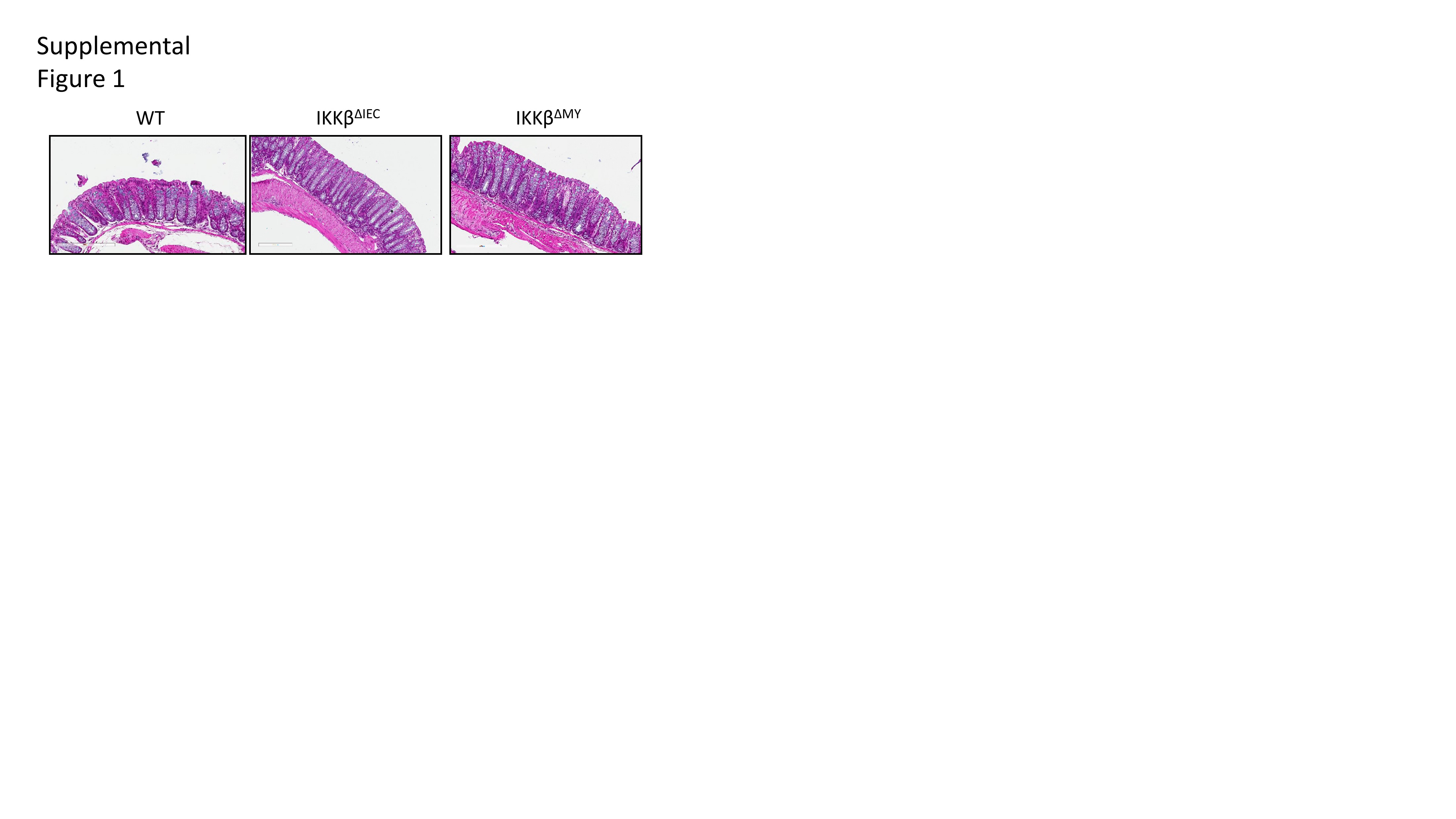

Supplement: Supplemental Figure 1 — Non-infected mice do not exhibit enhanced colonic histopathology. Paraffin-embedded colons were sectioned and stained with hematoxylin and eosin in order to visualize and score the pathology present in each sample. Representative images from non-infected mice are presented. n = 3/group. [file Image_1.jpg]

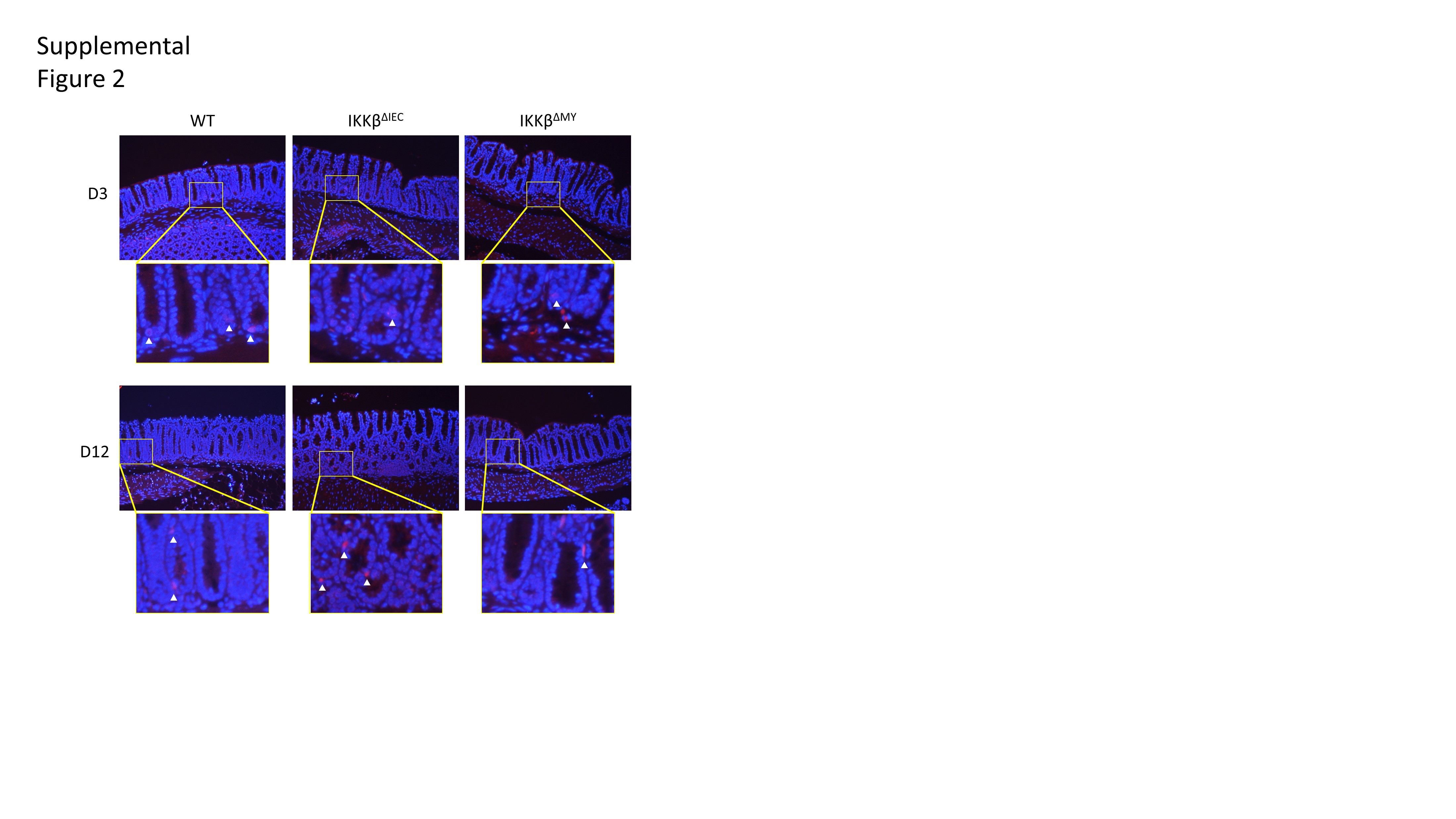

Supplement: Supplemental Figure 2 — Classical p65 expression is increased during C. rodentium challenge. Paraffin-embedded colons were sectioned and stained for phosphorylated p65 (red) and counterstained with DAPI (blue). n = 3/group. [file Image_2.jpg]

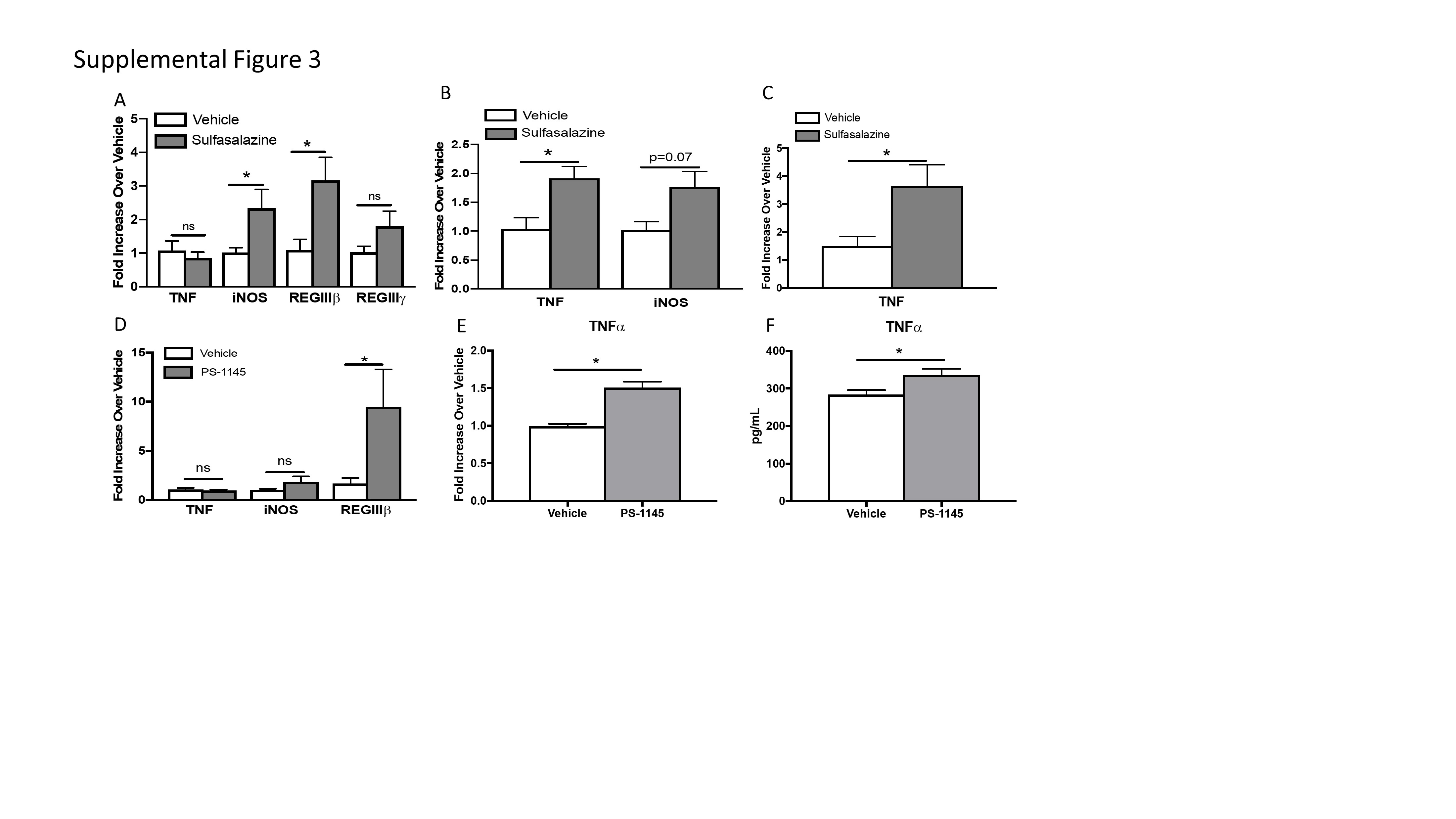

Supplement: Supplemental Figure 3 — Sulfasalazine and PS-1145 leads to altered gene expression in colonic epithelial cells and macrophages. (A) CMT-93 colonic epithelial cells. (B) RAW 264.7 macrophages and (C) CD11b+ ex vivo cultured splenocytes were inoculated with sulfasalazine (50 μm) overnight (CMT-93 and RAW 264.7) or 2 h (CD11b+). (A) Sulfasalazine enhanced baseline inflammatory (iNOS) and antimicrobial gene expression (RegIIIβ) in cultured CMT-93 cells *p < 0.05. Sulfasalazine treatment also increased TNF-α gene expression in (B) RAW 264.7 macrophages (*p < 0.05) and (C) CD11b+ monocytes (*p < 0.05). (D) CMT-93 colonic epithelial cells. (E,F) RAW 264.7 macrophages were treated with PS-1145 (10 μm) overnight. (D) PS-1145 significantly increased baseline RegIIIβ gene expression in CMT-93 cells *p < 0.05. (E) PS-1145 treatment significantly increased TNF-α expression in RAW 264.7 cells *p < 0.01. (F) TNF-α protein levels were also significantly increased by PS-1145 treatment in RAW 264.7 macrophages *p = 0.05. [file Image_3.jpg]
